# Vaginal repaired cesarean section diverticulum is beneficial in women with two prior cesarean sections

**DOI:** 10.1186/s12905-020-00940-8

**Published:** 2020-04-23

**Authors:** Yizhi Wang, Jiarui Li, Husheng Wang, Xipeng Wang

**Affiliations:** Departments of Gynecology and Obstetrics, Xin Hua Hospital Affiliated with Shanghai JiaoTong University School of Medicine, 1665 Kong Jiang Road, Shanghai, 200092 China

**Keywords:** Cesarean section diverticulum, Thickness of remaining muscular layer, Duration of menstruation, Vaginal repair

## Abstract

**Background:**

The aim of this study was to evaluate the effect of vaginal repair in patients with cesarean section diverticulum (CSD) who had one or two previous cesarean sections (CSs).

**Methods:**

From January 2012 to December 2014, 248 women with CSD underwent vaginal repair surgery in Shanghai First Maternity and Infant Hospital. These included 193 women with one previous cesarean section and 55 women with two previous cesarean sections. Excision and suture of CSD was performed through a vaginal approach. The duration of menstruation, the length, width and depth of the CSD and thickness of the remaining muscular layer (TRM) were evaluated before and after surgery by transvaginal three-dimensional (3D) color Doppler ultrasound.

**Results:**

A total of 221 (89.11%) women were followed-up for more than 3 months, and 168 (67.74%) women were followed-up for more than 6 months. There were significant differences in the average duration of menstruation (7.77 ± 2.05 and 8.02 ± 2.06 days VS 13.99 ± 3.71 days), the average size of CSD (5.54*9.19*5.60 and 5.75*9.04*6.18 mm VS 7.99*12.43*6.62 mm) and the TRM (7.61 ± 2.52 and 7.60 ± 3.00 mm VS 2.51 ± 1.02 mm) after surgery compared with those figures before surgery. The results of this study reveal that vaginal repair could shorten the duration of menstruation and improve anatomical defects (*P* < 0.05). Moreover, there was no significant difference in the effect of clinical repair between women with one or two previous cesarean sections (*P* > 0.05).

**Conclusion:**

In CSD patients, the clinical effectiveness of vaginal repair was equivalent between women with one or two previous cesarean sections.

## Background

The rate of cesarean section (CS) has increased over the last few decades [1, 2]. In 2008, 64.1% of urban women received cesarean section deliveries [3]. Compared with vaginal delivery, cesarean section is associated with a three- to six-fold higher risk of severe complications, such as intermenstrual bleeding, chronic pelvic pain, and the risk of secondary infertility, such as scar pregnancy, uterine rupture, placenta previa and accreta, all of which are now more common. All of these complications are associated with a new term in gynecologic disease. In a recent study, 64.5% of women who underwent cesarean section had cesarean section diverticulum (CSD), which causes abnormal uterine bleeding and other symptoms, within 6–12 weeks after surgery [4]. More than other complications, cesarean section scar ectopic pregnancies and CSD increase the risk of hemorrhoea or uterine rupture, which can threaten both the mother and her fetus’s lives [5, 6].

CSD is associated with two primary complications: abnormal uterine bleeding, which mainly presents as a longer duration menstruation period [7], and the thickness of the remaining muscular layer (TRM). Among these two complications, longer menstrual bleeding is a serious problem for many women. Additionally, using sanitary towels over a long period of time can lead to vaginitis and influence their social activities.

No guidelines are available for the treatment of CSD. In China, three main treatments have been proposed in recent years. One is to use traditional Chinese medicine to accommodate the menstrual period; however, this has a poor effect in shortening the menstrual period and has no effect on the muscular layer. The second method is to take oral contraceptives or receive placement of the Levonorgestrel intrauterine system (Mirena). However, not all women have a good response to these two therapies. Additionally, once the patient withdraws from the treatment, the symptoms of abnormal uterine bleeding return, and this therapy does not enhance the TRM. The third treatment is surgery. At present, there are three choices of operation methods: laparoscopic operation, hysteroscopy and vaginal repair [8, 9].

In a previous study, we showed that vaginal repair of CSD can improve the symptoms of postmenstrual spotting and anatomically correct its scars [10]. The purpose of this study was to compare the effects of vaginal repair between women with one or two previous cesarean sections.

## Methods

### Patients

We conducted a observational cohort study of data obtained in 248 patients with CSD who underwent vaginal repair between January 2012 and December 2014 in Shanghai First Maternity and Infant Hospital. In all, 193 of the women had one previous cesarean section and were included in Group A, while 55 of the women had two previous cesarean sections and were included as Group B. This study was approved by the Ethics Committee of Shanghai First Maternity and Infant Hospital affiliated with Tongji University (KS1512) and conducted in accordance with the Declaration of Helsinki. All patients signed written informed consent to participate in this study.

### Vaginal repair

All patients underwent vaginal repair at 7 to 14 days after menstruation. All procedures were conducted under epidural anesthesia and general anesthesia, and patients were placed in the lithotomy position. All surgical procedures reported in the current series were performed by the same surgeon. The only difference that we found was that some severe tissue adhesion was observed during the surgical procedure in patients who had two previous cesarean sections.

### Evaluation and follow-up

**Fig. 1:**
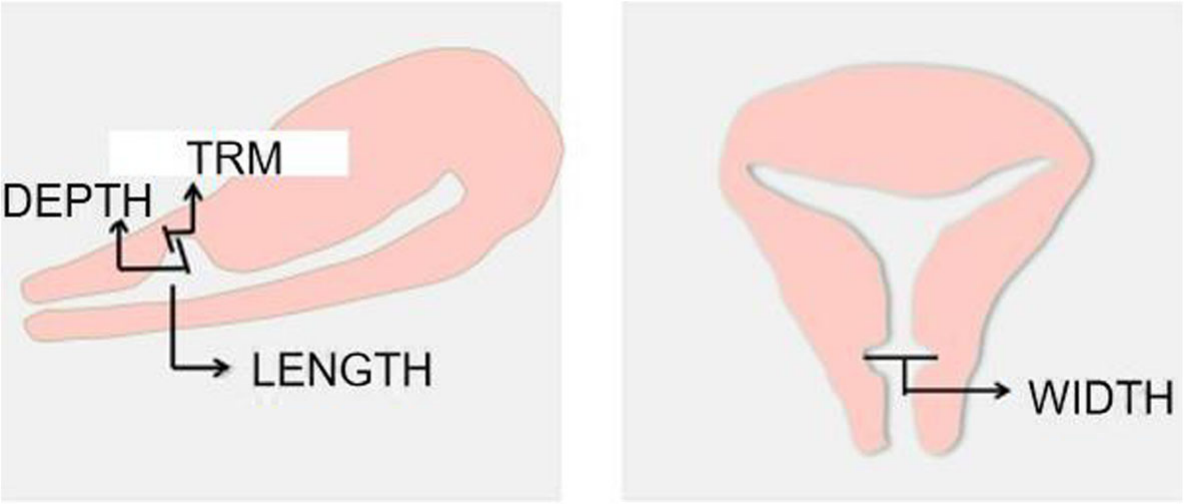
Ultrasonographic assessment of the cesarean section scar

We recorded all patients’ ages and their duration of menstruation before the surgery, and all of the patients were examined by transvaginal three-dimensional (3D) color Doppler ultrasound before the fourteenth day of menstruation to record their size of CSD as well as the TRM. TRM was measured from the interface between the uterine and the bladder wall to the bottom of the CSD. Length and depth were measured in sagittal plane and width were measured in transverse plane where the axis of the cervical canal can be demonstrated in relation to the lower segment and the uterine fundus.(Fig. 1) [11, 12]. After the surgery, the average temperature and the counts of leukocytes and hemoglobin in peripheral blood collected on the first day after surgery were evaluated. Additionally, the hospitalization duration and medical expenditures of the patients were also included in our study. At 3 and 6 months after surgery, the patients returned to the clinic provide data on the duration of their menstrual period. We then examined their TRM and the size of CSD by transvaginal ultrasound (TVU).

### Statistical analysis

Student-Newman-Keuls test and Bonferroni method were used to assess intergroup differences in the size of CSD, TRM and the duration of the menstrual period before and after surgical treatment. All of these data are expressed as the mean ± standard deviation (SD) or number (percentage). The outcomes of patients in different groups were compared using a completely random design ANOVA. *P* < 0.05 was considered statistically significant. Statistical analysis was performed using SPSS software version 18.0 (IBM, Chicago, IL, USA) and Systat Sigma Plot version 12.5 (Systat Software Inc., San Jose, CA, USA).

## Results

As shown in Table 1, a total of 248 patients were hospitalized and underwent vaginal repair. Of these, 193 had one and 55 had two previous cesarean sections. The average age was 32.64 ± 3.84 years old (*P* > 0.05). There was no significant difference in the duration of the menstrual period between before and after cesarean section (P > 0.05). Women with two prior cesarean sections suffered more severe symptoms with longer abnormal uterine bleeding. Before vaginal repair, the TRM of those who had two cesarean sections was higher than that found in women who had one prior cesarean section (3.17 ± 1.56 mm) (*P* < 0.05). There were no significant differences in hospitalization duration, medical expenditures incurred by inpatients, and leukocyte and hemoglobin counts after the surgery.

**Table 1:** Characteristics of Patients in the Cohorts

	Group A (*n* = 193)	Group B (*n* = 55)	*P* value
Age (y)	32.48 ± 3.67	32.80 ± 4.00	0.27
Duration of menstruation before CS (days)	6.13 ± 1.10	6.27 ± 1.06	0.56
Duration of menstruation after CS (days)	13.99 ± 3.71	15.05 ± 2.86	0.049
TRM before CS (mm)	2.51 ± 1.02	3.17 ± 1.56	0.005
Hospital stay (days)	6.53 ± 1.14	6.85 ± 0.95	0.14
Medical expenditure of inpatients (CNY)	11,243.50 ± 2581.30	11,142.84 ± 2261.77	0.59
Leukocyte after VR (10^9/L)	8.18 ± 2.30	8.02 ± 2.20	0.89
Hemoglobin after VR (g/L)	105.73 ± 12.04	103.68 ± 9.78	0.07

*CS* cesarean section, *TRM* thickness of remaining muscular layer, *VR* vaginal repair, *CNY* Chinese Yuan

According to the ultrasound data, a total of 223 women (89.92%) had visible CSD (Table 2). However, at 3 months after the surgery, only 63 and 18 of the patients who had one and two prior cesarean sections, respectively, still had CSD. At 6 months after vaginal repair, these numbers decreased to 45 (23.32%) and 12 (21.82%), respectively. Of those who still had CSD, the size of the CSD was significantly smaller than before the surgery (*P* < 0.05). However, there was no significant difference between the two groups (*P* > 0.05).

**Table 2:** The size of CSD before and after VR

	Group A (*n* = 193)	Group B (*n* = 55)
Size of CSD before VR (n, %)	7.99*12.43*6.62 (172, 89.12%)	7.76*11.71*6.20 (51, 92.73%)
Size of CSD at 3 months after VR (n, %)	5.54*9.19*5.60 (63, 32.64%)	6.22*10.17*5.89 (18, 32.73%)
Size of CSD at 6 months after VR (n, %)	5.75*9.04*6.18 (45, 23.32%)	5.42*11.58*5.08 (12, 21.82%)

*CSD* cesarean section diverticulum, *VR* vaginal repair

The duration of menstruation and the TRM after vaginal repair had both significantly improved after the surgery (P < 0.05). At 3 months after the surgery, there was no obvious difference between the groups in the TRM (Table 3). At 6 months postoperative (Table 3), 130 of the women (67.36%) who had one prior cesarean section and 38 of the women (69.10%) who had two prior cesarean sections returned to the clinic for evaluation. They all showed significant improvement in the duration of menstruation as well as the TRM. The average duration of menstruation had decreased from 14.52 ± 3.29 to 8.00 ± 2.19 days (*P* < 0.05). Nevertheless, there was no clear difference in the number of bleeding days between the two groups of patients (*P* > 0.05).

**Table 3:** The follow-up statistics of 3 and 6 months after VR

	Group A (*n* = 130)	Group B (*n* = 38)	P value
Average Duration of menstruation at 3 months after VR (day)	7.77 ± 2.05	8.06 ± 2.11	0.87
Average TRM at 3 months after VR (mm)	7.61 ± 2.52	8.04 ± 2.58	0.81
Average Duration of menstruation 6 months after VR (day)	8.02 ± 2.06	7.89 ± 2.32	0.44
Average TRM after VR at 6 months after VR (mm)	7.60 ± 3.00	8.22 ± 2.41	0.01

*TRM* thickness of remaining muscular layer, *VR* vaginal repair

## Discussion

In this study, we retrospectively analyzed outcomes in 248 patients who underwent vaginal repair surgery between January 2012 and December 2014. In all, 193 of them had one prior cesarean section, while 55 had two.

Recent analyses have suggested that while the optimal global cesarean section rate is almost 20%, attempts to reduce cesarean section rates in developed countries have not worked [13]. In China, the rate is approximately 35% [14], and the incidence of acquired diverticulum ranges from 4 to 30% and is mainly caused by poor healing following a cesarean section scars [7, 15].

Our country has enforced a two-child policy in recent years. Thus, many women have a cesarean section before they desire to have a second child. In these CSD patients, infertility is more common because the accumulated blood degrades the quality of sperm and the cervical mucus [16]. However, because the remaining muscular layer is thinner as a result of CSD, the risk of uterine rupture is significantly higher. Finally, with the promotion of the two-child policy, women who previously underwent one cesarean section are now more likely to experience two cesarean sections. Some of these women may experience abnormal uterine bleeding, which mainly presents as a longer duration menstruation period, compared to what had occurred before the cesarean section. Therefore, due to its long-term complications, CSD has recently received attention from more doctors.

Oral contraception is considered a conservative management option to treat CSD patients who have had two cesarean sections. One study showed that oral contraceptives improved patient symptoms by decreasing the volume of menstruation [17]. However, considering the ages of the affected patients, the use of oral contraceptives is controversial because of its potential risk of vein thrombosis [18].

Operative methods include hysteroscopy, laparoscopy and vaginal repair. Studies have indicated that compared to other surgical methods, vaginal repair for CSD is a minimally invasive procedure that allows good exposure and accurate resection [10, 17, 19, 20]. It also clearly shortened the duration of menstruation and significantly increased the distance between the CSD and the serosa [21]. Additionally, transvaginal repair may be a more cost-effective and convenient surgical approach for the management of patients with previous cesarean scar defects [19].

For patient with two prior cesarean sections, the main objectives are to shorten the duration of menstruation and improve quality of life. However, regarding the clinical effectiveness after vaginal surgery, whether women with two prior cesarean sections achieve better outcomes following this procedure has remained unclear.

Xu HY et al. found that repeated cesarean section is a risk factor for poor efficacy of scar repair, whether performed by laparoscopic surgery or transvaginal surgery (OR 9.75, 95%CI 2.30–41.36, 0.002) [22]. Nevertheless, in our study, we found that the duration of menstruation was significantly shorter after surgery in both groups (*P* < 0.05). However, there was no significant difference between the two groups (*P* > 0.05).

Another main symptom of CSD is a thinner remaining muscular layer. Compared to the data obtained before surgery, at the 3-month and 6-month follow-ups, there were significant differences in the size of the CSD as well as the TRM (P < 0.05). After vaginal repair, the average TRM was higher in group B than in group A. Hence, women with two prior cesarean sections achieved better outcomes than were achieved by those with one prior cesarean section. Finally, no complications, such as incomplete healing of the scar and bladder injury, were reported in the two groups.

In addition, there was an unknown problem. Before vaginal repair, the TRM was clearly higher in women with two prior cesarean sections than in those with one. According to some researchers, the cesarean section technique (i.e., single- or double-layer closure, whether or not a bladder flap is created, and closing of the peritoneum) plays an important role in niche development [23], and it has been proposed that placing continuous, nonlocking absorbable sutures in two layers without undue tightness (constricting/devascularizing) of the sutures is likely to result in good healing of uterine scar [24]. More research is needed to elaborate this point.

There are some limitations to our study. First, the sample size was limited in this paper, especially with regard for women with two prior cesarean sections. Second, the patients were seen for follow-up visits only after 3 and 6 months, and we need more follow-up data, particularly after 6 months, to determine long-term clinical effectiveness.

## Conclusions

In summary, our findings indicate that vaginal repair is an appropriate and effective method for the anatomical repair of CSD that shortens the duration of menstruation. This technique is equally effective in women who have had one and two cesarean sections. Because cesarean delivery is associated with increased long-term morbidity, the decision to perform cesarean section should always be carefully considered.

However, further studies performed on a larger scale and with longer follow-up times will certainly be needed to confirm our findings.

## Data Availability

The datasets generated and analysed during the current study are not publicly available due to related study still being in progress but are available from Yizhi Wang on reasonable request.The authors agreed to provide copies of the appropriate documentation if requested.

## Abbreviations

CS: Cesarean section
CSD: Cesarean section scar diverticulum
MRI: Magnetic resonance imaging
OC: Oral contraception
TRM: Thickness of the remaining muscular layer
VR: Vaginal repair
